# Phenotypes of a female patient with novel de novo frameshift ARX variant identified by whole-exome sequencing: a case report

**DOI:** 10.1097/MS9.0000000000000197

**Published:** 2023-02-07

**Authors:** Kristy Iskandar, Elisabeth S. Herini, Agung Triono, Marissa L. Hadiyanto, Andika P. Nugrahanto, Gunadi

**Affiliations:** a Department of Child Health; b Genetics Working Group, Faculty of Medicine, Public Health and Nursing; c Pediatric Surgery Division, Department of Surgery, Faculty of Medicine, Public Health and Nursing, Universitas Gadjah Mada/Dr. Sardjito Hospital, Yogyakarta, Indonesia.

**Keywords:** *ARX* variant, female patient, neurodevelopmental disorder, remarkable pleiotropy phenotypes, whole-exome sequencing

## Abstract

Variants in the *aristaless-related homeobox* (*ARX*) gene cause a diverse spectrum of phenotypes of neurodevelopmental disorders (NDD) in male patients. This article describes the role of genetic testing using whole-exome sequencing (WES) in detecting a novel de novo frameshift variant in the *ARX* gene in a female patient with autism, seizure, and global developmental delay.

**Case presentation:**

A 2-year-old girl with frequent seizures, global developmental delay, and autistic features was referred to our hospital. She was the second child of consanguineous non-affected parents. She had a high forehead, mildly prominent ears, and prominent nasal root. A generalized epileptiform discharge was noted in her electroencephalography. Brain MRI revealed corpus callosum agenesis, cerebral atrophy, and a left parafalcine cyst. The WES result showed a likely pathogenic variant identified as a novel de novo deletion in exon 4 of the *ARX* gene, which creates a frameshift variant. The patient is on dual therapy of antiepilepsy drugs, physiotherapy, speech therapy, occupational therapy, and oral motor exercises.

**Clinical discussion:**

Variants in the *ARX* gene can result in various phenotypes in males transmitted from asymptomatic carrier females. However, several reports showed that the *ARX* variants might cause phenotypes in females with milder symptoms than affected males.

**Conclusion:**

We report a novel de novo ARX variant in an affected female with a NDD. Our study confirms that the *ARX* variant might cause remarkable pleiotropy phenotypes in females. Moreover, WES could help to identify the pathogenic variant in NDD patients with diverse phenotypes.

Highlights

- The X-linked *aristaless-related homeobox* (ARX) gene variant is an rare neurodevelopmental disease (NDD) with various phenotypic spectrums.
- We describes a female child with autism, global developmental delay, seizure, and agenesis of corpus callosum associated with a novel de novo frameshift variant of the *ARX* gene.
- In developing countries with limited resources, whole-exome sequencing (WES) plays a crucial role in identifying patients with no specific disease symptoms.

## Introduction

The *ARX* gene, which contains five exons and encodes a 562 amino acid protein, plays an essential role in the early development and formation of the brain^1^. There are phenotypic spectrums of *ARX* variants ranging from hydranencephaly and lissencephaly (OMIM 300215); Proud syndrome (OMIM 300004); infantile spasms without brain malformations or developmental and epileptic encephalopathy 1 (OMIM 308350); intellectual developmental disorder (OMIM 300419); to Partington syndrome (OMIM 309510)^2^. Several previous studies showed that various variants in the *ARX* gene, including large deletions, frameshifts, nonsensical variants, splice site variants, and missense variants, revealed remarkable pleiotropic phenotypes. It consists of a nearly continuous series of developmental disorders, starting from hydranencephaly, and alters stepwise to lissencephaly, to corpus callosum agenesis without other anomalies, and to an overlapping syndromes series with obviously normal brain anatomy^3^,^4^.

A variant in the *ARX* gene on the X chromosome usually results in families with affected males passing it down through carrier females. However, there has recently been an increase in reported *ARX* variants in females, with a generally milder phenotype than in affected males^1^. We identified a novel de novo frameshift variant in the *ARX* gene using the WES approach in a female patient with autism, seizures, and global developmental delay phenotype. Our case report has been reported in line with the 2013 CARE guideline^5^.

## Case presentation

### History

A 2-year-old girl was referred to our hospital due to frequent seizures, difficulty interacting with her surroundings, and global developmental delay. Seizures, which first occurred at 10 months, were recognized when she suddenly stared blankly into space with circumoral cyanosis for a few seconds. She had these episodes one to two times per month for 1–2 min. She was the second child of consanguineous healthy parents and had two sisters (4-year-old and 1-year-old), both with normal development. The patient was born by spontaneous vaginal delivery at 39 weeks of gestation to a 26-year-old mother with normal birth weight and head circumference.

The patient tended to like repetitively clapping, hitting her head, ignoring others, being unable to follow commands, and having difficulty making eye contact and interacting with her surroundings. She was screened for autism spectrum disorder (ASD) based on the Modified Checklist for Autism in Toddlers Revised (M-CHAT-R) Questionnaire completed by her parents. Her score of 16 showed a high risk of ASD, and the diagnosis of ASD was confirmed by two pediatric neurologists using the *Diagnostic Statistical Manual of Mental Disorders, Fifth Edition* (DSM-5) criteria. Developmental delay was diagnosed and classified using the Denver Developmental Screening Test (DDST). At 2 years old, she could only walk two to three steps, could not duplicate lines, expressed her desire by crying, and could not speak any clear words yet.

### Physical examination

**Figure 1:**
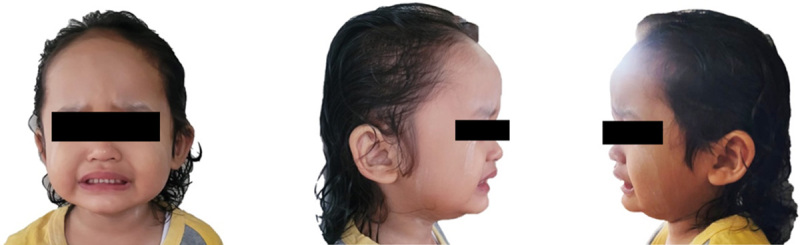
The patient had a high forehead, mildly prominent ears, and prominent nasal root.

**Figure 2:**
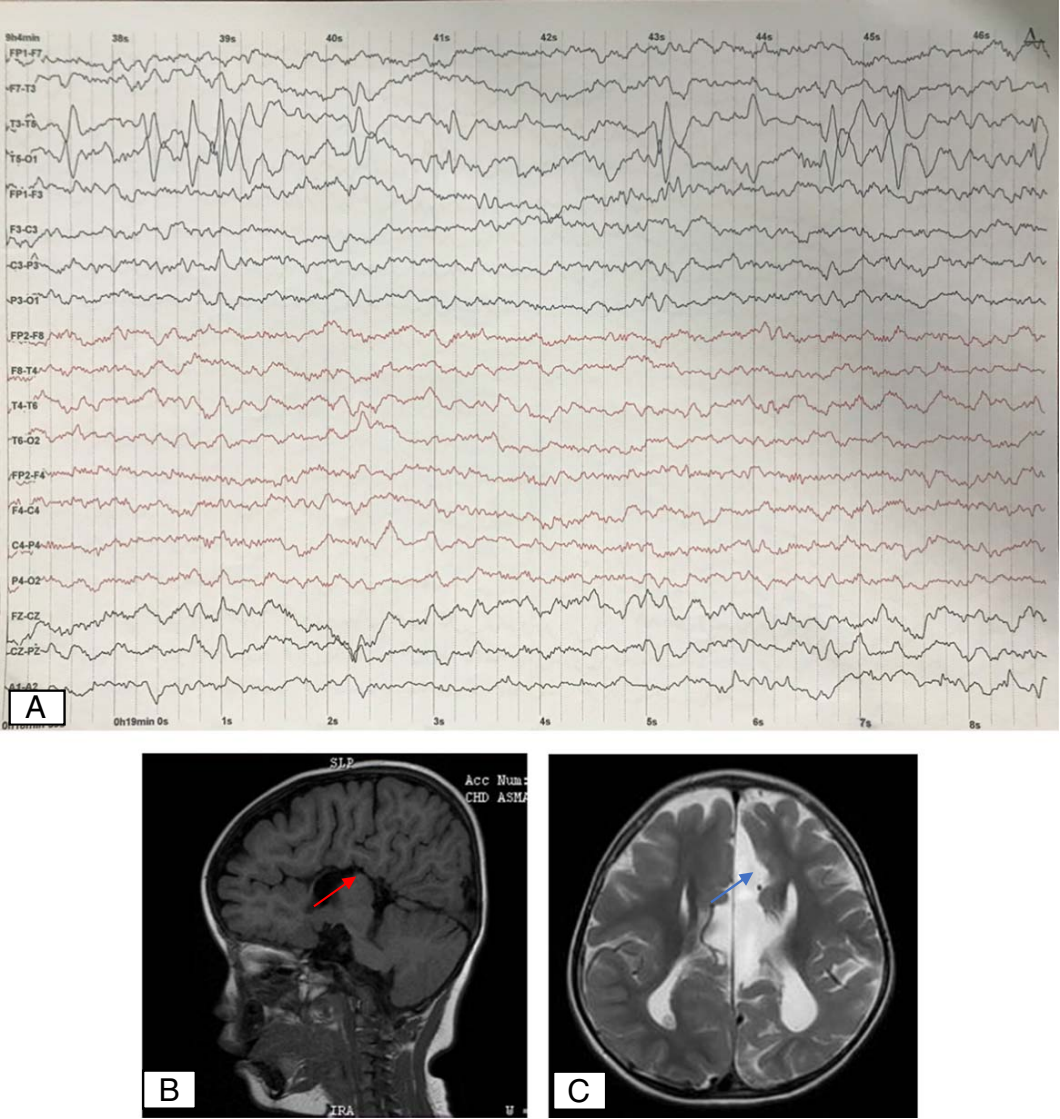
(A) Electroencephalography showed generalized epileptiform discharge on left temporo-occipital. (B) Sagittal T1-weighted image sequence. (C) Axial T2-weighted image sequence brain MRI showed corpus callosum hypoplasia (red arrow), left parafalcine cyst (blue arrow), and cerebral atrophy.

She had a normal head circumference, a high forehead, mildly prominent ears, and a prominent nasal root (Fig. 1). Neurological examination showed positive clonus and increased physiological reflexes in all extremities. Cardiac, pulmonary, gastrointestinal, and genitourinary examinations showed no abnormality. Electroencephalography (EEG) (Fig. 2A) showed generalized epileptiform discharge. Brain MRI showed corpus callosum agenesis, cerebral atrophy, and a left parafalcine cyst (Figs. 2B and C). The patient is on valproic acid (35 mg/kg body weight/day) and phenobarbital (4 mg/kg body weight/day) therapy. In the last year, the patient only experienced one seizure per year with a less than 1-min duration after using these drugs. The patient also routinely does physiotherapy, speech therapy, occupational therapy, and oral motor exercises.

### Genetic analysis

**Figure 3:**
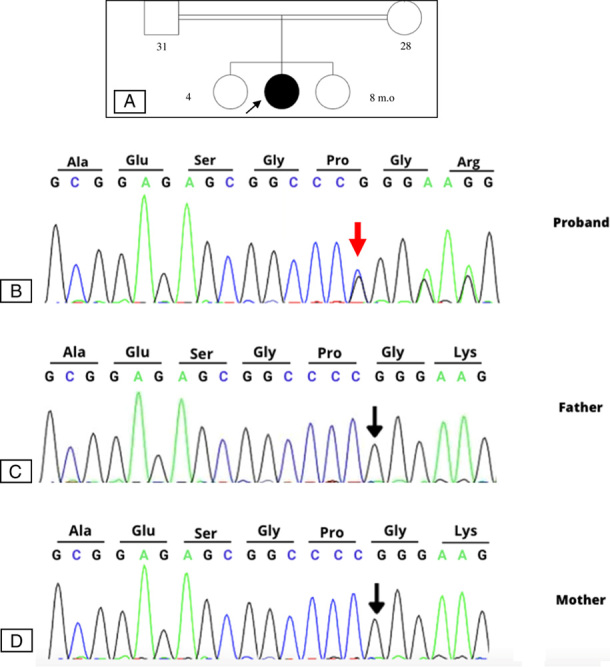
(A) The family’s pedigree showed the consanguineous marriages of the parents of the patient. DNA sequence chromatogram of exon 4 of ARX gene in the proband (B), father (C), and mother (D). Sanger analysis confirmed the presence of the heterozygous ARX mutation (NM_139058.3:c.1206del; NP_620689.1:p.Pro403ArgfsTer60) in proband’s DNA, while the normal sequence was confirmed in the unaffected father and mother.

Blood samples were collected after written informed consent. The WES process was conducted using the protocol from Richards *et al.* 6 and Seo *et al.* 7. WES and Sanger sequencing were performed in an accredited laboratory, 3Billion (Seoul, Korea). The raw genome sequencing data analysis, including alignment to the GRCh37/hg19 human reference genome, variant calling, and annotation, was performed using open-source bioinformatics tools and custom software. The automatic variant interpretation software, EVIDENCE, was developed in-house to prioritize variants based on the American College of Medical Genetics and Genomics (ACMG) guideline^8^ and each patient’s phenotype. The WES result showed a deletion in exon 4 in the *ARX* gene located on chromosome X:25025469,c.1206del (p.Pro403ArgfsTer60), which creates a frameshift variant. The variant-generated stop codon is quite close (59 bp) to the exon-exon junction, with exon 5 being the terminal exon in ARX. It might cause a loss of function due to nonsense-mediated mRNA decay and does not produce a truncated protein containing a C-terminal missense peptide that escapes nonsense-mediated mRNA decay. Based on the variant score using the ACMG guidelines, this variant is classified as likely pathogenic. This novel variant was submitted to ClinVar by 3Billion^9^. Sanger sequencing was performed to confirm the patient’s genotypes and the inheritance pattern. The patient was shown to have a de novo variant (Fig. 3).

## Discussion

Here, we report a female child with autism, global developmental delay, seizures, and brain malformation associated with a novel de novo frameshift variant of the *ARX* gene. The patient had a 1 bp deletion in exon 4 with cytogenetic location Xp21.3 (Pro403fs), which caused a frameshift variant. Variants in the X-linked *ARX* gene have recently been shown to cause mental retardation with a broad spectrum of neurological disorders^10^.

*ARX* is a homeobox-containing gene that is expressed in the developing nervous system. *ARX* is essential for the proliferation of neural progenitors and the migration of excitatory cortical neurons, according to a study that involved human brain samples and animal models^11^,^12^. It also affects the migration and differentiation of GABAergic inhibitory interneurons^12^. Consistently, male *ARX*-deficient mice had smaller brains and thinner cerebral cortical layers^11^.

Previously, affected individuals were predominately male; the female phenotype was defined almost exclusively through identifying mothers or other female relatives of male probands. The phenotype of the affected female varies, as do changes in the corpus callosum, which is sometimes associated with mild intellectual disability and/or epilepsy and/or anxiety and depression. Moreover, Mattiske *et al.* 1 showed that in familial cases, affected females have a milder phenotype than affected males. To the best of our knowledge, only a few cases of *ARX* mutations affecting females were reported (Table 1). Our patient had a phenotype similar to several de novo cases, including global developmental delay, autism, and seizures.

**Table 1:** Clinical features of affected females with the *ARX* variants

cDNA/protein changes	Variant type	Development	Seizure characteristics	Brain malformation	Additional features	References
c.869C^>^A p.S290X	Nonsense	Severe DD	EIEE (onset 1 month age)	Microcephaly	CVI, dystonia	Kwong *et al.* 12
c.1459delA p.Thr487GlnfsX5.	Deletion (frameshift)	GPD	EIEE (onset 3 months age)	–	Autistic features, significantly ataxic, strabismus, congenital hip dislocation	Bettella *et al.* 13
c.1465delG p.A489Pfs^∗^3	Deletion (frameshift)	GDD	IS (onset 4 months age)	Cysts	Hypotonia, torticollis, plagiocephaly, small bilateral epicanthal fold with mildly low set ear with slightly over folded helices	Wallerstein *et al.* 14
c.1206del (p.Pro403ArgfsTer60)	Deletion (frameshift)	GDD	Absence and focal seizure (onset 10 months age)	Callosum agenesis, cerebral atrophy, and a left parafalcine cyst	High forehead, mildly prominent ears, and prominent nasal root	This report

CVI, cortical visual impairment; DD, developmental delay; EIEE, early infantile epileptic encephalopathy; GDD, global developmental delay; GPD, global psychomotor delay; ID, intellectual disability; IS, infantile spasm.

There are phenotypic spectrums of *ARX* variants dependent on the underlying genetic mechanism^2^,^13^. Coman *et al.* 14 reported an infant with multisystem disorders, primarily neurological and gastrointestinal malformations, caused by substitution variants in exon 4. Troester *et al.* 15 reported a male with mental retardation and developmental delay was found with 24 bp deletions in exon 2. Mattiske and colleagues reported that a family with multiple affected individuals with a 10-year-old female proband showed learning difficulties, mild intellectual disability, and seizures. The genetic result of an insertion variant in exon 2 of the *ARX* gene appears to cause a frameshift variant^1^. Similar clinical manifestations were observed in our patient with developmental delay, autism, and seizures, with evidence of a frameshift variant in exon 4.

Global developmental delay or intellectual disability is frequently found in patients with *ARX* variants^16^–^18^, including in our patient. Another clinical finding in other studies was hypothalamic dysfunction with deficient body temperature control, chronic diarrhea, and poor feeding, which was not observed in our patient^3^,^14^,^19^. A case report by Bonneau *et al.* 20 reported craniofacial abnormalities, including a prominent forehead, large anterior fontanel, long and smooth philtrum, thin lips, and mild micrognathia. Some of those clinical features were also found in our patient, who has a high forehead, mildly prominent ears, and prominent nasal root.

Intractable epilepsy has been reported in patients with *ARX* gene variants^1^,^21^. Epilepsy in patients with the *ARX* gene variants was associated with GABAergic function abnormalities and impairment in forebrain cerebral cortical interneuron function^3^,^19^. However, our patient did not have any intractable seizures, which was also reported by Mattiske *et al.* 1 that the female proband had a milder phenotype of the *ARX* variants. At 10 months old, our patient had absence and focal seizures one to two times per month with only valproic acid therapy, which was then reduced to once a year after administration of phenobarbital. This is also in accordance with a patient with X-linked ambiguous genitalia who also became seizure-free after being treated with phenytoin and phenobarbital^22^. The EEG results found in patients with X-linked *ARX* gene variants were continuous bilateral, multifocal epileptic discharges and a lack of normal background rhythms^14^,^22^. This is consistent with our patient’s EEG patterns.

MRI findings usually found in patients with an *ARX* variant were lissencephaly, agyria or pachygyria and agenesis of the corpus callosum^3^,^23^. A case report of X-linked lissencephaly with abnormal genitalia caused by a frameshift variant of the *ARX* gene had MRI findings similar to those of our patient, including the agenesis corpus callosum, enlarged ventricles, colpocephaly, and also no lissencephaly found^1^. There has been a wide range of neurological phenotypes caused by variants in the *ARX* gene, implying that many different degrees of structural cerebral malformation can be found in radiological examinations^3^. The prognosis for LISX2 is poor, mainly when it occurs in male patients. Most patients die in the first 18 months of life, and the survival rate is up to 4 years of age. After receiving dual therapy of antiepilepsy drugs and rehabilitative therapy, our patient can walk several steps and has decreased seizure frequency.

To the best of our knowledge, this is the first case of the *ARX* variant in Indonesia. This might be due to the fact that genetic testing is still expensive and not routinely performed in our daily practice. WES might be one of the solutions for detecting illnesses with various genetically diverse symptoms. WES provides a superior alternative to gene panel testing at a comparable cost for children with suspected complex monogenic phenotypes^24^.

## Conclusions

Here, we report a novel de novo variant in the *ARX* gene in an affected female with a NDD. Our study confirms that the *ARX* variant might cause remarkable pleiotropy phenotypes in females. Moreover, WES could help to identify the pathogenic variant in NDD patients with diverse phenotypes, particularly in developing countries with limited resources. These findings will have a beneficial impact on genetic counseling for families.

## Ethical approval

The Medical and Health Research Ethics Committee of the Faculty of Medicine, Public Health and Nursing, Universitas Gadjah Mada/Dr Sardjito Hospital ruled the study exempt from approval because this study was a case report (KE/0695/05/2022).

## Consent

Written informed consent was obtained from the patient for the publication of this case report and accompanying images. A copy of the written consent is available for review by the Editor-in-Chief of this journal on request.

## Sources of funding

This study was supported by the Ministry of Research and Technology/National Research and Innovation Agency Republic of Indonesia (RISTEK/BRIN) (PDUPT to KI). The funding body did not influence the study design, data analysis, data interpretation, and manuscript writing.

## Authors’ contributions

K.I., A.T., and E.S.H. wrote, designed the study, and edited the manuscript. Gunadi supervised and reviewed the manuscript. M.L.H. and A.P.N. wrote the manuscript, collected and analyzed the clinical data. All the authors read and approved the final manuscript.

## Conflicts of interest disclosure

The authors declare that they have no financial conflict of interest with regard to the content of this report.

## Research registration unique identifying number (UIN)

Not applicable.

## Guarantor

Elisabeth S. Herini.

## Provenance and peer review

Not commissioned, externally peer-reviewed.
